# Two roads for oncolytic immunotherapy development

**DOI:** 10.1186/s40425-019-0515-2

**Published:** 2019-02-01

**Authors:** Howard L. Kaufman, Praveen K. Bommareddy

**Affiliations:** 1 Division of Surgical Oncology, Massachusetts General Hospital, 55 Fruit Street, Warren 401, Boston, MA 02114 USA; 2 Replimune, Inc., Woburn, MA USA; 3 Rutgers Graduate School of Biomedical Sciences, New Brunswick, NJ USA

## Abstract

Oncolytic viruses are an emerging class of immunotherapy agents for cancer treatment. In this issue of JITC, Machiels et al. reports early phase data from an oncolytic adenovirus given by intravenous (IV) administration. While this may allow easy access to metastatic lesions, there is limited data supporting the therapeutic effectiveness of this approach. Further studies should include assessment of viral replication in tumor tissue and consider comparative trials using IV and intratumoral delivery to fully optimize oncolytic immunotherapy.

Oncolytic immunotherapy (OI) is a new approach for cancer treatment that utilizes live or heat-inactivated viruses that preferentially replicate in tumor cells, induce immunogenic cell death (ICD), and initiate host anti-tumor immunity [1]. Globally, there are three approved oncolytic viruses, an adenovirus (H101) for the treatment of advanced head and neck cancer in China, Rigvir, an oncolytic reovirus approved for the treatment of advanced melanoma in Estonia, Latvia, Poland and Belarus, and most notably, talimogene laherparepvec (T-VEC), an oncolytic herpes simplex virus, type 1 (HSV-1) approved for the treatment of advanced melanoma in the United States, Europe and Australia [1, 2]. The ability of oncolytic viruses (OVs) to induce ICD and recruit lymphocytes into the tumor microenvironment has generated considerable interest in using OVs as part of combination regimens designed to enhance the therapeutic effectiveness of cancer immunotherapy. Indeed, recent clinical reports have supported a significant therapeutic benefit when T-VEC was used in combination with immune checkpoint inhibitors in melanoma [3, 4]. As a class, OVs can be differentiated from other forms of immunotherapy in being live, replicating agents that can be amplified in vivo (at least until neutralized by the host immune system), having a tolerable safety profile (largely causing low grade constitutional symptoms and local injection site reactions), and to date, most have been delivered by intra-tumoral (IT) injection [5]. While IT administration allows maximal delivery of high viral titers to tumors, bypasses systemic neutralization and prevents premature clearance, IT delivery can cause logistical and biosafety concerns especially when used in busy ambulatory or in-patient clinical settings. Further, many patients harbor metastatic disease at sites not easily palpable, and require localization via interventional imaging or surgical exposure, which may be challenging for OVs that require repeated administrations over time. The potential to delivery OVs by IV administration theoretically allows for viral distribution to tumors at any site and precludes the need for additional training and interventional procedures associated with IT delivery.

In this issue of JITC, Machiels et al. report the results of a Phase I clinical trial using an intravenously (IV) delivered chimeric adenovirus, enadenotucirev, in patients with advanced epithelial solid tumors [6]. This virus was developed specifically on the basis of its blood stability and potential suitability for IV administration. In the study, 61 patients were treated initially in a standard dose escalation design with viral doses of 1 × 10^10^–1 × 10^13^ viral particles (vp) given on days 1, 3 and 5, and subsequent patients treated in an expansion cohort or using various schedules of virus delivery. Enadenotucirev treatment was associated with typical OV adverse events with the most common treatment-emergent grade 3 or greater adverse events being hypoxia, lymphopenia and neutropenia. The trial established a dose of 3 × 10^12^ vp as the maximum tolerated dose, and an additional 9 patients were treated in an expansion cohort at this dose with further patients enrolled to evaluate weekly and every three-week dosing schedules. Although no objective responses were seen, the authors concluded that the study supports further clinical investigation of IV enadenotucirev in combination with other agents. Although the study provides important safety and dosing information, does it truly support the potential therapeutic effectiveness of IV administration for OVs and what other data would be helpful to better evaluate the impact of IV administration in lieu of objective tumor regression?

In a prior trial of enadenotucirev, 17 patients with a variety of primary solid tumors received a single IV dose of virus and 8–15 days later underwent tumor resection [7]. In this study, tumor specimens had evidence of virus by IHC and confirmed by PCR assay. In the Machiels et al. study, evidence for active OV accumulation at the tumor site was evaluated in one patient with metastatic colorectal cancer who had an abdominal wall metastasis available for biopsy 39 days after treatment. In this patient, who had received enadenotucirev in every three-week schedule, biopsy revealed extensive necrosis and viral infection of tumor cells was seen by immunohistochemistry and viral replication confirmed by qPCR of viral DNA sequences, consistent with the data from the earlier trial. While PCR is a highly sensitive assay for viral sequences, confirmation of live viral particles and on-going replication cannot be established. Additional studies of local viral protein expression or recovery and plaque assay of live viruses with determination of viral titers pre- and post-treatment might be more informative. The issues with IV delivery of any OV includes considerable dilution in the systemic circulation and the likelihood of premature neutralization through serum anti-viral immunoglobulins or other serum proteins, especially after multiple infusions. Thus, confirmation that effective virus is delivered to metastatic lesions is necessary when evaluating IV delivery and may differ depending on the viral species, host immune response and history of prior viral exposure and features of the local tumor microenvironment that may impede effective viral delivery to sites of tumor growth.

To date, there have been only a few published reports of IV delivery of OVs and the key findings from these studies are summarized in Table 1. In addition to the current report by Machiels et al. in this issue of JITC, as mentioned above, enadenotucirev was previously evaluated in a phase 1 mechanism of action study in 17 patients with solid tumors scheduled for primary resection were treated with intravenous virus (1 × 10^12^ vp on days 1, 3 and 5) followed by operation on days 8–25 [7]. This study is the only one to include a comparator cohort of patients with colorectal cancer treated by IT injection, although this was limited to only five subjects. The investigators assessed the presence of virus within tumor by immunohistochemical (IHC) staining for nuclear viral hexon protein expression and qPCR for viral DNA sequences. Virus was detected in tumor tissue by both injection methods, but while virus was more commonly confirmed in IV-treated tumors, intense hexon IHC staining (> 8-%) was more common in IT-treated lesions. The authors, however, did not report viral titers from the tumor sites.

**Table 1:** Selected studies of intravenous oncolytic virus delivery

Study	OV species	Tumors targeted	Sample size	Dose range	Treatment schedule	Intratumoral OV analysis	Adverse events
Machiels et al. [6]	Adenovirus	Epithelial adeno Carcinomas	61	1 × 10^10^–1 × 10^13^ vp	Days 1, 3 and 5 weekly and every 3-week schedule	One patient with colorectal cancer abdominal wall metastasis sample was + by IHC and qPCR 39 days after treatment	Hypoxia, lymphopenia, neutropenia
Nemunaitis et al. [8]	Adenovirus (ONYX-015)	Solid tumors metastatic to lung	10	2 × 10^10^–2 × 10^13^ vp	Weekly in 21 day cycle	qPCR and IHC Virus seen in one tumor biopsy	Flu-like symptoms, transient transaminitis
Hamid et al. [9]	Adenovirus	Metastatic colorectal cancer	18	2 × 10^12^ vp	Every 2 weeks	One autopsy patient with tumor at the root of the mesentery by PCR and IHC	flu-like symptoms, chills, fatigue and lethargy
Rudin et al. [10]	Seneca valley virus	Small cell lung cancer and carcinoid tumors	30	10^7^ – 10^11^ vp	Single dose	qPCR and IHC one autopsy-derived tumor had + IHC for virus	Flu-like symptoms
Park et al. [11]	Vaccinia virus-GM-CSF	Treatment-refractory colorectal cancer	15	1 × 10^6^ – 3 × 10^7 pfu^	Every 14 days	Plaque assay on plasma and throat swabs	Flu-like symptoms
Downs-Canner et al. [12]	Vaccinia virus	Advanced colorectal or other solid cancers	11	3 × 10^8^–3 × 10^9^ pfu	Single dose	qPCR Plaque assay detected 2.5 × 103 pfu in one patient	fever, chills, abdominal pain, nausea, vomiting, fatigue
Garcia et al. [13]	Adenovirus type 5	Metastatic melanoma	12	1 × 10^13^ vp	Single infusion	qPCR Viral DNA was only detected in patients treated with doses > 3.3 × 10^11^	flu- like syndrome fever, chills, neutropenia
Garcia-Carbonero, et al. [7]	Adenovirus	Solid adenocarcinomas	17 12 by IV 5 by IT inj.	1 × 10^12^ vp	Days 1, 3 and 5 followed by tumor resection	Virus hexon protein by IHC found in 10 patients > 80% nuclear staining seen in 21.1% of IT-inj. and 9.4% for IV-inj. Tumor specimens	None
Mell et al. [14]	Vaccinia virus	Head and neck cancer	19	3 × 10^8^–3 × 10^9^ pfu	Day 3 Day 3 and 8 Days 3, 8, 15 and 22 Radiation 33–35 fractions Cisplatin on days 1, 22 and 43	qPCR+ in 5 patients (range 4–409 copies/mg) Virus (2.0 × 10^3^ pfu) detected in tongue tumor in 1 patient at 7 days	Rigors, fever, fatigue, rash, hypotension, mucositis, nausea, vomiting
Samson et al. [15]	Reovirus	Brain tumors	9	1 × 10^10^ TCID_50_	Single one-hour infusion	IHC for reovirus ó3 capsid protein was low in 6/9 tumors igTEM + in 9/9 ISH+ 8/9 qPCR+ in 4/7	Lymphopenia, flu-like symptoms

Abbreviations: *igTEM* immunogold transmission electron microscopy, *inj.* injection, *IT* intratumoral, *IV* intravenous, *pfu* plaque-forming units, *qPCR* quantitative polymerase chain reaction assay, *TCID* tissue culture infective dose, *vp* viral particle

Several other OVs have been tested in early phase clinical trials with similar results (Table 1). ONYX-015 was a replication competent E1B-deleted adenovirus that selectively replicates in tumor cells harboring p53 mutations. A small ten patient study of ONYX-015 in patients with advanced solid tumors metastatic to the lung was conducted with IV delivery weekly in 21 day cycles [8]. Virus was detected in plasma by PCR analysis and one patient had intratumoral virus detected by IHC and PCR. ONYX-015 was also delivered by IV delivery every two weeks in a phase 2 study of 18 patients with treatment refractory colorectal cancer [9]. In this study, 36% of patients had viral DNA detected by PCR in plasma 72 h after the last treatment and one patient who died of disease progression was found to have virus by IHC and PCR in the spleen and normal liver at autopsy with very low levels in tumor tissue. A phase I study of an oncolytic Seneca Valley virus was conducted in 30 patients, six with small cell lung cancer and 24 with carcinoid tumors [10]. The virus was given by a single IV delivery using a dose escalation design and viral titers in blood were seen at day 3 by PCR. In one patient who died tumor tissue was obtained and virus was detected by IHC. Pexa-Vec is a thymidine kinase gene-deficient oncolytic vaccinia virus encoding GM-CSF and was evaluated in 15 patients with treatment-refractory colorectal cancer with increasing doses given by IV delivery every two weeks [11]. Infectious viral titers were detected in plasma two hours after cycle 1 and 30 min after cycle 4, as well as low doses in throat swabs 5–8 days after treatment, but no tumor was assessed for presence of virus. Using a Western Reserve strain of vaccinia virus, a phase 1 study in 11 patients with advanced colorectal cancer were treated with a single IV dose in a dose escalation design [12]. Virus was detected in blood on days 3 and 8 while two patients had virus found in tumor tissue by PCR analysis. ICOVIR-5 is a modified oncolytic adenovirus that has a deletion of the E1A region and has an RGD sequence inserted at the HI loop of the fiber knob to target integrins and assist in tumor cell entry [13]. Twelve advanced cutaneous and uveal melanoma patients were treated with a single dose of ICOVIR-5 at increasing doses. The authors reported that 2 of 3 patients at the higher doses had virus detected by qPCR assay. Across all these studies adverse events were largely low grade constitutional symptoms and no objective clinical responses were seen.

GL-ONC1 is a modified oncolytic vaccinia virus, which has been administered intravenously in combination with chemoradiation therapy in patients with primary locoregional head and neck carcinomas [14]. In a phase 1 dose finding trial, 19 patients were treated and the OV was well tolerated with the most common adverse events being grade 1–2 rigors, fever, fatigue, and rash. More serious grade 3 adverse reactions included hypotension, mucositis, nausea, and vomiting. The investigators detected OV by qPCR assay in 4 patient resected tumors and 1 patient demonstrated live virus in a tongue neoplasm 7 days after receiving the first dose of virus. In another trial using an oncolytic orthoreovirus, patients with high-grade glioma and CNS metastases from solid tumors were treated with a single IV administration followed by resection [15]. The authors reported a tolerable safety profile with an increase in T cell infiltration and local interferon and PD-1/PD-L1 expression following OV delivery. In this study they examined viral presence through multiple assays on post-resection tumor specimens and reported that reovirus ó3-capsid protein expression was detected at low levels in 6 of 9 tumor specimens. Using in situ hybridization of reovirus RNA was detected in 8 tumors and using an immunogold transmission electron microscopy technique, virus was detected in all 9 specimens. In addition, the investigators utilized a qPCR assay but found viral copies in only 4 of the 9 tumor biopsies highlighting the limitations of PCR analyses.

Collectively, the clinical data thus far suggest that while IV delivery of OVs across several different viral species is safe and well tolerated, detection of high titers at tumor sites has not been clearly demonstrated by IV delivery. Viral biodistribution represents a significant challenge as many OVs will be able to infect both normal and neoplastic cells. Although most OVs undergo abortive replication in normal cells, these cells which may include easily accessible immune cells, can harbor viral particles and prevent effective delivery to the tumor site. In addition, rapid clearance of virus through both pre-existing and induced antibody titers, as well as through binding of some viruses to serum proteins, may further limit delivery of virus to tumor sites. This issue may become more pronounced over time in patients treated with multiple viral infusions. Thus, how can we move forward? Further clinical investigation should include sensitive and accurate assessment of viral titers recovered from tumor sites in patients treated with OVs in early phase clinical trials. As shown by the limited data in Table 1, quantitative PCR assay may not be reliable in confirming live virus. An attempt to evaluate neutralizing anti-viral antibody titers is also important to better understand how this mechanism may limit viral delivery to tumors. Further consideration of clinical studies that compare IV and IT delivery would also be important for understanding the nuances and potential of each approach. The final arbiter of success will likely be evidence of tumor regression and, while the current studies focused on IV administration are early and not designed to evaluate therapeutic activity, to date no evidence for clinical responses have been observed. Prior to embarking on large combination clinical trials, it may be prudent to understand the biology of OV delivery in more detail to better optimize dosing, schedules and routes of administration.

As Robert Frost wrote,

“Two roads diverged in a wood, and I—.

I took the one less traveled by,

And that has made all the difference.”
