# Correction: *Two roads for oncolytic immunotherapy development*

**DOI:** 10.1136/s40425-019-0515-2corr1

**Published:** 2021-06-16

Kaufman HL, Bommareddy PK. Two roads for oncolytic immunotherapy development. *J Immunother Cancer* 2019;7:26. doi: 10.1186/s40425-019-0515-2

This correction notice is to note that second sentence of the first paragraph should be corrected from

‘Globally, there are three approved oncolytic viruses, an adenovirus (H101) for the treatment of advanced head and neck cancer in China, Rigvir, an oncolytic reovirus approved for the treatment of advanced melanoma in Estonia, Latvia, Poland and Belarus, and most notably, talimogene laherparepvec (T-VEC), an oncolytic herpes simplex virus, type 1 (HSV-1) approved for the treatment of advanced melanoma in the United States, Europe and Australia.^1 2^

To

‘Globally, there are three approved oncolytic viruses, an adenovirus (H101) for the treatment of advanced head and neck cancer in the People’s Republic of China; Rigvir, an oncolytic echovirus approved for the treatment of advanced melanoma in some Eastern European countries; and most notably, talimogene laherparepvec (T-VEC), an oncolytic herpes simplex virus, type 1 (HSV-1) approved for the treatment of advanced melanoma in the United States, Europe, Australia and Israel.’^1 2^

